# GLP-1 Receptor Agonists or SGLT2 Inhibitors and Nonarteritic Anterior Ischemic Optic Neuropathy

**DOI:** 10.1001/jamanetworkopen.2026.9917

**Published:** 2026-04-30

**Authors:** Taeyoung Choi, Ziyad Al-Aly, Yan Xie

**Affiliations:** 1 Clinical Epidemiology Center, Research and Development Service, VA St Louis Health Care System, St Louis, Missouri; 2 Veterans Research and Education Foundation of St Louis, St Louis, Missouri; 3 Division of Pharmacoepidemiology, Clinical Epidemiology Center, Research and Development Service, VA St Louis Health Care System, St Louis, Missouri; 4 Department of Medicine, Washington University School of Medicine, St Louis, Missouri; 5 Nephrology Section, Medicine Service, VA St Louis Health Care System, St Louis, Missouri; 6 Institute for Public Health, Washington University in St Louis, St Louis, Missouri; 7 Division of Public Health Sciences, Department of Surgery, Washington University School of Medicine, St Louis, Missouri

## Abstract

This cohort study evaluates the 3-year risk of nonarteritic anterior ischemic optic neuropathy among veterans with type 2 diabetes who initiated glucagon-like peptide-1 receptor agonists (GLP-1RAs) or a sodium-glucose cotransporter-2 (SGLT2) inhibitor.

## Introduction

Glucagon-like peptide-1 receptor agonists (GLP-1RA) are increasingly being used for various indications.^1,2^ Pharmacovigilance reports suggest a disproportionate signal of ocular adverse events and visual impairment among GLP-1RA users, and emerging population-based studies raise concern about an increased risk of nonarteritic anterior ischemic optic neuropathy (NAION).^3,4,5^ However, findings remain inconsistent,^5,6^ and it remains unclear whether GLP-1RA use is specifically associated with NAION rather than other optic disorders.

## Methods

For this cohort study, we leveraged the nationwide electronic health records of the US Department of Veterans Affairs (VA) to emulate a target trial to compare the 3-year risk of NAION among veterans with type 2 diabetes who initiated GLP-1RAs or sodium-glucose cotransporter-2 inhibitors (SGLT2i) between January 1, 2017, and December 31, 2024 (eTable in Supplement 1). The date of analysis was November 28, 2025.

NAION diagnosis was defined by *International Statistical Classification of Diseases and Related Health Problems, Tenth Revision (ICD-10)* code H47.01*. We validated all* ICD-10*–identified cases using clinical text documentation, yielding a positive predictive value of 84.70% (95% CI, 83.10–86.20). We evaluated alternative definitions including NAION diagnosis by an eye care specialist, repeated NAION diagnoses, and specialist diagnosed NAION with repeated diagnosis (eMethods in Supplement 1).

We balanced the GLP-1RA and SGLT2i groups through propensity score–based inverse probability weighting to estimate the intention-to-treat average treatment effect within the GLP-1RA group. Weighted Fine and Gray subdistribution hazard models, accounting for death as a competing risk, were used to estimate the 3-year cumulative incidence, cumulative incidence difference (CID), and cumulative incidence ratio (CIR). A 95% CI for the CIR excluding 1 was considered statistically significant.

To evaluate the specificity of the association between GLP-1RA vs SGLT2i on risk of NAION, we additionally examined several optic disorders including diabetic retinopathy, macular degeneration, retinal vascular occlusion, and optic neuritis. We also assessed the frequency of ophthalmology or optometry visits during follow-up (eMethods in Supplement 1).

We reported the study following both the Strengthening the Reporting of Observational Studies in Epidemiology (STROBE) and Transparent Reporting of Studies Emulating a Target Trial (TARGET) reporting guidelines. The study was approved with a waiver of informed consent by the VA St. Louis Health Care System institutional review board because the study involved minimal risk and could not be performed without the waiver. Data were analyzed using SAS Enterprise Guide version 8.3 (SAS Institute Inc).

## Results

The study included 588 168 participants, with 139 546 who initiated GLP-1RAs (125 870 [90.20%] male, 27 394 [19.63%] Black, 106 829 [76.56%] White, and 5323 [3.81%] other races) and 448 622 who initiated SGLT2is (427 626 [95.32%] male, 90 876 [20.26%] Black, 338 624 [75.48%] White, and 19 122 [4.26%] other races). After weighting, age and BMI (calculated as weight in kilograms divided by height in meters squared) were similar between groups (mean [SD] age, 65.33 [10.98] vs 65.02 [10.95] years and mean [SD] BMI, 35.77 [7.01] vs 36.02 [7.12] for the GLP-1RA and SGLT2i groups, respectively). Baseline characteristics for the cohort are shown in the Table.

**Table.** Baseline Characteristics of Recipients of Glucagon-Like Peptide-1 Receptor Agonists (GLP-1RA) and Sodium-Glucose Cotransporter-2 Inhibitors (SGLT2i) Before and After Weighting

Baseline characteristics	Unweighted			Weighted		
	Participants, No. (%)		SMD^a^	Participants, No. (%)		SMD^a^
	GLP-1RA (n = 139 546)	SGLT2i (n = 448 622)		GLP-1RA (n = 139 546)	SGLT2i (n = 448 622)	
Age, mean (SD), y	65.33 (10.98)	67.94 (10.68)	−0.24	65.33 (10.98)	65.02 (10.95)	0.03
Sex						
Male	125 870 (90.20)	427 626 (95.32)	−0.20	125 870 (90.20)	402 015 (89.61)	0.02
Female	13 676 (9.80)	20 996 (4.68)	0.20	13 676 (9.80)	46 607 (10.39)	−0.02
Race^b^						
Black	27 394 (19.63)	90 876 (20.26)	−0.02	27 394 (19.63)	88 523 (19.73)	−0.003
White	106 829 (76.56)	338 624 (75.48)	0.03	106 829 (76.56)	343 151 (76.49)	0.002
Other^c^	5323 (3.81)	19 122 (4.26)	−0.02	5323 (3.81)	16 948 (3.78)	0.002
Smoking status						
Never	56 901 (40.78)	169 910 (37.87)	0.06	56 901 (40.78)	184 599 (41.14)	−0.01
Former	58 752 (42.10)	194 250 (43.30)	−0.02	58 752 (42.10)	186 972 (41.68)	0.01
Current	23 893 (17.12)	84 462 (18.83)	−0.04	23 893 (17.12)	77 051 (17.18)	−0.001
BMI, mean (SD)^d^	35.77 (7.01)	32.84 (6.30)	0.44	35.77 (7.01)	36.02 (7.12)	−0.04
Estimated glomerular filtration rate, mean (SD), mL/min/1.73m^2^	75.01 (22.18)	74.69 (20.41)	0.02	75.01 (22.18)	76.03 (21.75)	−0.05
Systolic blood pressure, mean (SD), mmHg	132.86 (16.56)	133.71 (17.26)	−0.05	132.86 (16.56)	132.83 (16.49)	0.002
Diastolic blood pressure, mean (SD), mmHg	76.01 (9.84)	76.14 (9.90)	−0.01	76.01 (9.84)	76.02 (9.86)	−0.001
HbA_1c_, mean (SD), %	8.61 (2.43)	8.23 (1.66)	0.19	8.61 (2.43)	8.65 (1.82)	−0.02
HbA_1c_ within 1 y, mean (SD)	8.53 (1.67)	8.13 (1.54)	0.25	8.53 (1.67)	8.58 (1.68)	−0.03
Low-density lipoprotein, mean (SD), mg/dL	83.49 (35.67)	83.11 (35.86)	0.01	83.49 (35.67)	83.58 (35.74)	−0.002
Cancer	6257 (4.48)	24 379 (5.43)	−0.04	6257 (4.48)	19 751 (4.40)	0.004
AKI	11 229 (8.05)	28 408 (6.33)	0.07	11 229 (8.05)	35 004 (7.80)	0.01
Hyperlipidemia	59 720 (42.80)	179 982 (40.12)	0.05	59 720 (42.80)	193 219 (43.07)	−0.01
Nonalcoholic fatty liver disease	6983 (5.00)	18 266 (4.07)	0.05	6983 (5.00)	23 726 (5.29)	−0.01
Peripheral artery disease	3003 (2.15)	9850 (2.20)	−0.003	3003 (2.15)	9884 (2.20)	−0.004
Stroke	3968 (2.84)	14 609 (3.26)	−0.02	3968 (2.84)	13 143 (2.93)	−0.01
Myocardial infarction	1544 (1.11)	8134 (1.81)	−0.06	1544 (1.11)	4907 (1.09)	0.001
Heart failure	12 196 (8.74)	59 167 (13.19)	−0.14	12 196 (8.74)	36 988 (8.24)	0.02
Cataract	39 566 (28.35)	126 922 (28.29)	0.001	39 566 (28.35)	126 913 (28.29)	0.001
Macular degeneration	8899 (6.38)	31 933 (7.12)	−0.03	8899 (6.38)	27 915 (6.22)	0.01
Diabetic retinopathy	21 480 (15.39)	52 403 (11.68)	0.11	21 480 (15.39)	69 451 (15.48)	−0.002
Diabetic neuropathy	43 544 (31.20)	108 355 (24.15)	0.16	43 544 (31.20)	144 654 (32.24)	−0.02
Diabetic nephropathy	18 883 (13.53)	48 583 (10.83)	0.08	18 883 (13.53)	58 269 (12.99)	0.02
Ocular surgery	4428 (3.17)	13 815 (3.08)	0.01	4428 (3.17)	13 851 (3.09)	0.01
Eye care visits or ophthalmology examination	89 221 (63.94)	271 669 (60.56)	0.07	89 221 (63.94)	288 732 (64.36)	−0.01
Metformin	93 123 (66.73)	302 990 (67.54)	−0.02	93 123 (66.73)	304 389 (67.85)	−0.02
Insulin	82 564 (59.17)	146 494 (32.65)	0.55	82 564 (59.17)	271 963 (60.62)	−0.03
DPP4i	28 882 (20.70)	84 283 (18.79)	0.05	28 882 (20.70)	100 401 (22.38)	−0.04
Sulfonylureas	46 024 (32.98)	151 744 (33.82)	−0.02	46 024 (32.98)	149 916 (33.42)	−0.01
Thiazolidinediones	8948 (6.41)	22 430 (5.00)	0.06	8948 (6.41)	30 829 (6.87)	−0.02

Abbreviations: AKI, acute kidney injury; BMI, body mass index; DPP4i, dipeptidyl peptidase-4 inhibitors; HbA_1c_, hemoglobin A_1c_; SMD, standardized mean difference.

SI conversions: To convert HbA_1c_ to proportion of total hemoglobin, multiply by 0.01; low-density lipoprotein to millimoles per liter, multiply by 0.0259.

^a^ SMD value range between −0.1 and 0.1 indicates balance was achieved.

^b^ Self-reported race information was collected from electronic health records and used in the study in accordance with the requirement by the funding agency (US Department of Veterans Affairs) and the Office of Management and Budget, which defines minimum standards for maintaining, collecting, and presenting data on race and ethnicity for all federal reporting agencies.

^c^ Other race included Alaska Native and American Indian, Asian or Native Hawaiian and Other Pacific Islander.

^d^ BMI calculated as weight in kilograms divided by height in meters squared.

Over 3 years of follow-up, the cumulative incidence of NAION was 39.07 (95% CI, 35.38-43.06) per 10 000 persons in the GLP-1RA group and 29.33 (95% CI, 26.34-33.06) per 10 000 persons in the SGLT2i group. Compared with SGLT2i, GLP-1RA use was associated with an increased risk of NAION (CID, 9.98; 95% CI, 3.48-14.03 per 10 000 persons at 3 years; CIR, 1.35; 95% CI, 1.11-1.51) (Figure).

**Figure.**
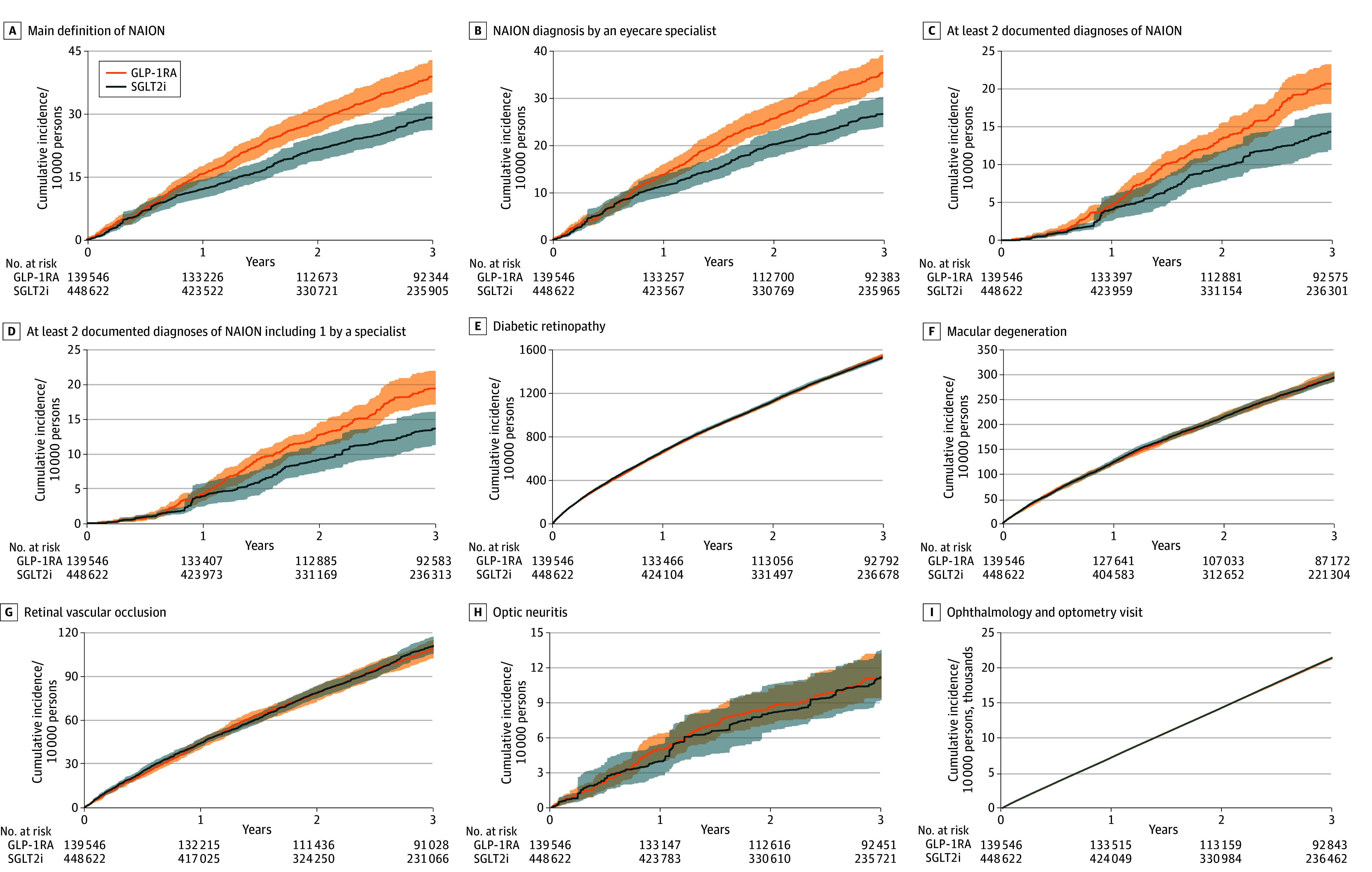
Survival Curves Showing Cumulative Incidence of Nonarteritic Anterior Ischemic Optic Neuropathy (NAION) and Other Optic Disorders in Glucagon-Like Peptide-1 Receptor Agonists (GLP-1RA) and Sodium-Glucose Cotransporter-2 Inhibitors (SGLT2i) Groups A, Cumulative incidence of NAION; outcome defined as at least 1 *International Statistical Classification of Diseases and Related Health Problems, Tenth Revision (ICD-10)* code H47.01* diagnosis in any setting. Cumulative incidence per 10 000 persons for GLP-1RA and SGLT2i; cumulative incidence difference and cumulative incidence ratio at 3 years are included. B through D, Cumulative incidence of alternative NAION definitions. B, Outcome defined by at least 1 *ICD-10* code H47.01* made by an ophthalmology or optometry specialist. C, Outcome defined by at least 2 NAION diagnoses more than 30 days apart in any clinical setting. D, Outcome defined by at least 2 NAION diagnoses more than 30 days apart, at least 1 of which was made by an ophthalmology or optometry specialist. E through H, Cumulative incidence of other optic disorders. I, frequency of ophthalmology and optometry visit.

The increased risk associated with GLP-1RA use was consistently observed across alternative NAION definitions, including NAION diagnosis by an eye-care specialist (CID, 8.73; 95% CI, 2.46-12.89; CIR, 1.34; 95% CI, 1.08-1.52), repeated NAION diagnoses (CID, 6.35; 95% CI, 2.40–9.65; CIR, 1.46; 95% CI, 1.14-1.76), and specialist diagnosed NAION with repeated diagnosis (CID, 5.91; 95% CI, 2.00-8.88; CIR, 1.43; 95% CI, 1.13-1.74) (Figure). We additionally evaluated other optic disorders to assess the specificity of the observed association with NAION. Compared with SGLT2i use, GLP-1RA use was not associated with risk of other ophthalmic disorders, including diabetic retinopathy (CIR, 1.01; 95% CI, 0.99-1.03), macular degeneration (CIR, 1.00; 95% CI, 0.97–1.05), retinal vascular occlusion (CIR, 0.98; 95% CI, 0.89-1.05), or optic neuritis (CIR, 1.00; 95% CI, 0.77-1.32). The frequency of ophthalmology or optometry clinic visits during follow-up was also similar between groups (CIR, 1.00; 95% CI, 0.99-1.00) (Figure).

## Discussion

In this large cohort study, GLP-1RA use was associated with a modestly increased risk of NAION compared with SGLT2i use. While the absolute risk remains low, the specificity of this finding may warrant heightened vigilance.

This study has limitations. The cohort was older and predominantly male. Residual confounding, selection bias, and outcome misclassification, including incomplete capture of events outside the VA, cannot be fully excluded.

## Notes

Supplement 1. eMethods. eReferences.

Supplement 2. Data Sharing Statement
