# Supplementary material for: GLP-1 Receptor Agonists or SGLT2 Inhibitors and Nonarteritic Anterior Ischemic Optic Neuropathy
Source: JAMA Netw Open. 2026 Apr 30;9(4):e269917. doi: 10.1001/jamanetworkopen.2026.9917 (PMC13133691; doi:10.1001/jamanetworkopen.2026.9917)
Supplement: Supplement 1. — eMethods. eReferences. [file jamanetwopen-e269917-s001.pdf]

Supplemental Online Content

Choi T, Al-Aly Z, Xie Y. GLP-1 receptor agonists or SGLT2 inhibitors and nonarteritic anterior ischemic optic neuropathy in type 2 diabetes. *JAMA Netw Open*. 2026;9(4):e269917.
doi:10.1001/jamanetworkopen.2026.9917

eMethods.

eReferences.

This supplemental material has been provided by the authors to give readers additional information about their work.

© 2026 Choi T et al. *JAMA Network Open*.

Target trial emulation

We designed a pragmatic target trial that would enroll participants who had no use of GLP-1RA or SGLT2i in the prior year, no contraindications to GLP-1RA or SGLT2i, and no history of NAION. Participants in the target trial would be randomly assigned to initiate GLP-1RA or SGLT2i and followed for up to three years to evaluate the effect of GLP-1RA initiation on the risk of NAION. We then emulated this target trial using data from the U.S. Department of Veterans Affairs electronic health records. The protocol describing the specifications of the target trial and its emulation is presented below.

eTable1. Specification and emulation of the target trial.

Protocol Components	Target Trial Protocol	Emulation
**Research question**	Among US veterans with type 2 diabetes who are eligible for both GLP-1 receptor agonists (GLP-1RA, intervention) and SGLT2 inhibitors (SGLT2i, active comparator), and who have no prior history of NAION, what is the risk of non-arteritic anterior ischemic optic neuropathy (NAION) after initiating GLP-1RA versus initiating SGLT2i?	Same as protocol
**Enrollment period**	The enrollment period is from January 1, 2017 to December 31, 2024.	Same as protocol
**Eligibility criteria**	Inclusion: US veterans who<br>• Had type 2 diabetes,<br>• Were VA health care system users (defined as having ≥2 encounters and ≥1 pharmacy utilization in the year before the enrollment date).<br>Exclusion:<br>• Had contraindication for GLP-1RA or SGLT2i (diabetic ketoacidosis, gastroparesis, malignant neoplasm of thyroid gland, multiple endocrine neoplasia type II, pancreatitis, severe hypoglycemia [hypoglycemia with coma or requiring hospitalization], or severe renal impairment [eGFR <30 mL/min/1.73 m², dialysis, or kidney transplant]) in the year before the enrollment date),	Same as protocol

© 2026 Choi T et al. *JAMA Network Open*.

	• Had prior use of GLP-1RA in the year before the enrollment date,<br>• Had prior use of SGLT2i in the year before the enrollment date,<br>• Enroll during hospitalization or emergency room visit<br>• Had a prior diagnosis of NAION	
**Treatment assignment**	Eligible participants would be randomized to initiate either a GLP-1RA (intervention) or an SGLT2i (active comparator). Individuals will be aware of the assigned treatment strategy.	We emulated random treatment assignment by applying inverse probability weighting.<br>Propensity scores used for weighting were estimated using six prespecified domains of baseline covariates as detailed in the Methods.<br><br>We empirically evaluated the emulated randomization of treatment by assessing covariate balance between treatment groups after weighting; standardized mean differences (SMDs) between -0.10 and 0.10 were taken as evidence of adequate balance and used as a proxy for successful randomization.
**Treatment initiation**	Participants received either (1) GLP-1RA (intervention) or (2) SGLT2i (comparator) on the date of treatment assignment.<br><br>The calendar date of the index dispensing is T_0.	Treatment initiation based on outpatient pharmacy records of release of the medication.
**Treatment strategy**	Participants initiate the medication to which they are assigned:<br><br>(1) Intervention: GLP-1RA.<br>(2) Comparator: SGLT2i.<br><br>Treatment is open-label and participants are aware of their assigned strategy.	Same as the protocol.
**Outcomes**	Incident diagnosis of NAION	Same as protocol.
**Follow-up**	Follow-up starts at treatment initiation (T_0). Participants will be followed until the earliest occurrence of outcome, death, administrative censoring on October 31, 2025, or at 3 years after T_0.	Same as protocol.
**Causal contrasts of interest**	Intention-to-treat (ITT) effect of initiating GLP-1RA versus initiating SGLT2i	Same as protocol.

© 2026 Choi T et al. *JAMA Network Open*.

**Analysis plan to estimate causal contrasts of interest**	The ITT effect would be estimated based on Fine and Gray subdistribution hazard model where death would be considered as competing risk in the model. Marginal cumulative incidence functions for each treatment group would be derived from the subdistribution hazards separately. The 3-year cumulative incidence per 10,000 person at 3 years, cumulative incidence difference, and cumulative incidence ratio would be then estimated based on the cumulative incidence function for each group.	Same as protocol.
**Causal estimand**	Among participants with type 2 diabetes in the VA system who are eligible to initiate GLP-1RA or SGLT2i and have no prior NAION, the intention-to-treat effect of initiating GLP-1RA versus initiating SGLT2i at baseline on the 3-year cumulative incidence of NAION, with death treated as a competing risk with administrative censoring at 3 years or October 31, 2025 , summarized as the average treatment effect among the treated expressed as the difference and ratio in 3-year cumulative incidence between groups.	Same as protocol.

© 2026 Choi T et al. *JAMA Network Open*.

Exposures

Treatment was defined as initiation of GLP-1RA in the outpatient setting. The active comparator was defined as initiation of SGLT2i in the outpatient setting. GLP-1RA agents included albiglutide (1.53%), dulaglutide (16.61%), exenatide (1.52%), liraglutide (22.35%), lixisenatide (0.01%), semaglutide (56.95%), and tirzepatide (1.03%). SGLT2i agents included empagliflozin (99.86%); the remaining agents (bexagliflozin, canagliflozin, dapagliflozin, ertugliflozin, and sotagliflozin) together accounted for 0.14%.

Date of treatment initiation (T_0) was defined based on release date of the initiated medication from outpatient pharmacy.

© 2026 Choi T et al. *JAMA Network Open*.

Outcomes

The primary outcome was incident NAION diagnosis, defined by at least one ICD-10 code H47.01*. To assess the accuracy of this ICD-based definition, we validated all ICD-identified cases using clinical text documentation. Among 1,761 ICD-identified cases, 1,492 were confirmed based on documentation, yielding a positive predictive value of 84.70% (83.10–86.20).

We additionally evaluated NAION using alternative definitions: NAION diagnosis by an eye-care specialist, defined by at least one ICD-10 code H47.01* made by an ophthalmology or optometry specialist. Repeated NAION diagnoses, defined by at least two NAION diagnoses more than 30 days apart in any clinical setting. Specialist diagnosed NAION with repeated diagnosis, defined by at least two NAION diagnoses more than 30 days apart, at least one of which is made by ophthalmology or optometry specialist.

To evaluate the specificity of the association between GLP-1RA vs SGLT2i on risk of NAION, we examined several additional optic disorders including diabetic retinopathy (ICD-10 code E08.3*, E09.3*, E10.3*, E11.3*, E13.3*), macular degeneration (ICD-10 code H35.31* and H35.32*), retinal vascular occlusion (ICD-10 code H34.*) and optic neuritis (ICD-10 code H46.*). These outcomes were selected because they are driven by pathophysiological mechanisms distinct from NAION. We also assessed the frequency of ophthalmology or optometry visits during follow-up to evaluate potential differences in ophthalmic surveillance between groups.

© 2026 Choi T et al. *JAMA Network Open*.

Covariates

The directed acyclic graphs (DAG) reflects assumed relationships between treatment, NAION, and measured covariates based on prior clinical and epidemiologic knowledge and was used to guide identification of potential confounders requiring adjustment (eFigure 1)[1-5].

eFigure 1. Directed acyclic graph

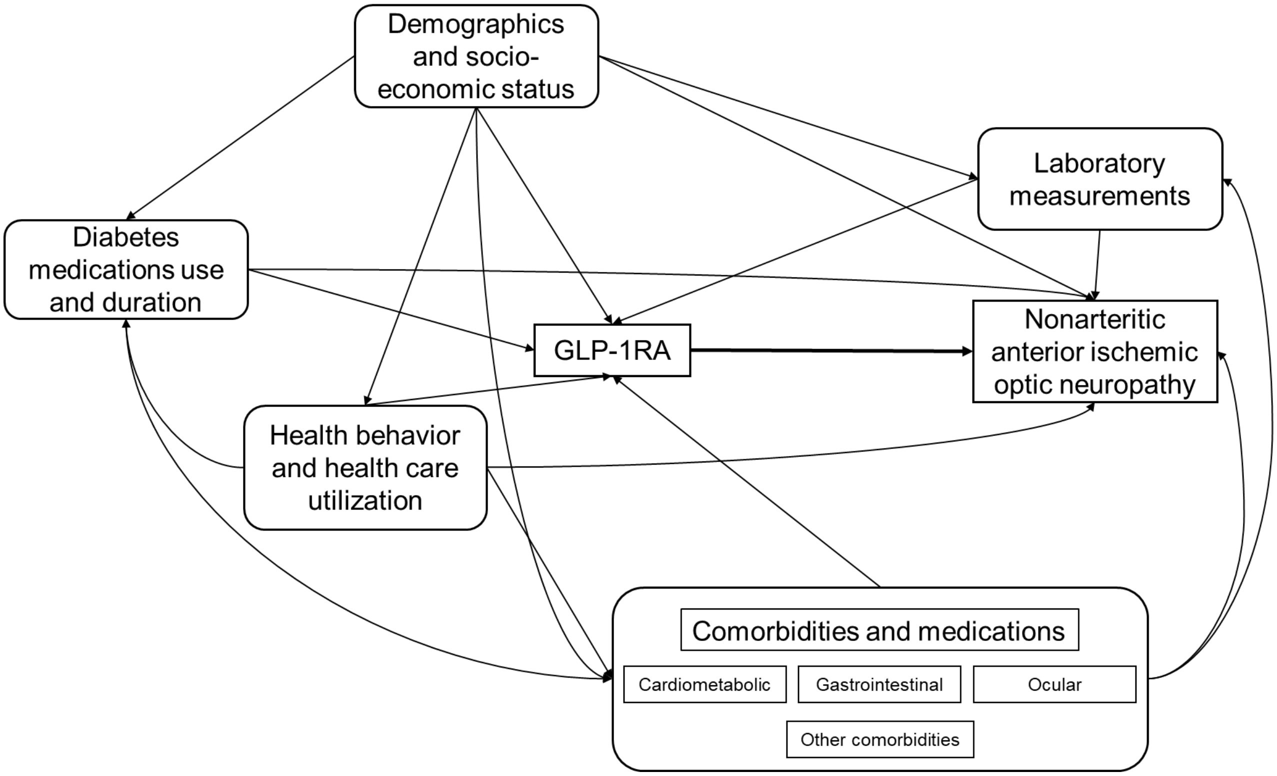

Covariates were measured within one year before enrollment date unless otherwise specified. When multiple records of same covariates existed, the one before and closest to the enrollment date was selected. Selected covariates comprised socio-demographic status including age, race (black, white, and other), sex, and area deprivation index; vital measurements including body mass index (BMI) and blood pressure; laboratory or physiological measurements including hemoglobin A1c (HbA1c) at baseline, average HbA1c within 1 year, and separately within 5 years before T_0, albuminuria, low-density lipoprotein (LDL), and estimated glomerular

© 2026 Choi T et al. *JAMA Network Open*.

filtration rate (eGFR); health behavior and health care utilization including smoking status (current, former, and never), number of inpatients and outpatients visits, number of blood panel tests, number of prescriptions, number of HbA1c measurements, number of surgeries, eye-care visits/ophthalmology exams, long-term care, community care and number of inpatient and outpatient visits from Medicare. We also adjusted the calendar week of enrollment since January 2017.

Additionally, we adjusted for history of antiplatelet and anticoagulant use, and history of diabetic medication use including metformin, insulin, dipeptidyl peptidase-4 inhibitor (DPP4i), sulfonylureas, and thiazolidinediones. We also adjusted duration of metformin, insulin, DPP4i, sulfonylureas, and thiazolidinediones use within 5 years before receiving diabetic medication.

We also adjusted for angiotensin converting enzyme inhibitors, angiotensin receptor blockers, calcium channel blockers, beta-blockers, diuretics, statin, PDE-5 inhibitors, bupropion, naltrexone, orlistat, phentermine, and topiramate.

For the comorbidities, our model included duration of diabetes from October 1999 till T_0, nausea, diarrhea, abdominal pain, dyspepsia, constipation, gastroesophageal reflux disease (GERD), fatigue, suicidal ideation, thyroid cancer, hypertension, chronic kidney disease (CKD), dementia, depression, peripheral artery disease (PAD), alcohol use disorder, hypoglycemia, cancer, human immunodeficiency virus (HIV), urine tract infection, acute kidney injury (AKI), hyperlipidemia, non-alcoholic fatty liver disease, sleep apnea, stroke, transient ischemic attack (TIA), atrial fibrillation, myocardial infarction, ischemic cardiomyopathy, angina, heart failure, non-ischemic cardiomyopathy, deep vein thrombosis, thyroid disorders, nutritional deficiencies, gastroparesis, diabetic retinopathy, diabetic neuropathy, diabetic nephropathy, obesity, bariatric surgery, ocular surgery, and diseases of the eye and adnexa using ICD-10 codes H00.*-H59.* (e.g., glaucoma, cataract, and macular degeneration).

Missing values including 3.86% BMI, 0.13 % diastolic and systolic blood pressure, 0.69 % eGFR, 3.72% HbA1c, 0.76 % LDL measurements were imputed based on predictive mean matching method where matching values were generated based on fully conditional specification with chained equations[6]. Continuous variables including age, eGFR, HbA1c, BMI, LDL, blood pressure, number of outpatient visits, number of prescriptions,

© 2026 Choi T et al. *JAMA Network Open*.

number of blood panel tests, number of HbA1c measurements, and calendar week of enrollment were transformed into restricted cubic splines with knots placed at 5th, 35th, 65th and 95th percentiles[7].

eReferences

1. Xie Y, Bowe B, Xian H, Loux T, McGill JB, Al-Aly Z. Comparative effectiveness of SGLT2 inhibitors, GLP-1 receptor agonists, DPP-4 inhibitors, and sulfonylureas on risk of major adverse cardiovascular events: emulation of a randomised target trial using electronic health records. *Lancet Diabetes Endocrinol*. Sep 2023;11(9):644-656. doi:10.1016/S2213-8587(23)00171-7
2. Xie Y, Bowe B, Gibson AK, McGill JB, Maddukuri G, Al-Aly Z. Clinical Implications of Estimated Glomerular Filtration Rate Dip Following Sodium-Glucose Cotransporter-2 Inhibitor Initiation on Cardiovascular and Kidney Outcomes. *J Am Heart Assoc*. Jun 2021;10(11):e020237. doi:10.1161/JAHA.120.020237
3. Xie Y, Bowe B, Gibson AK, McGill JB, Maddukuri G, Al-Aly Z. Comparative Effectiveness of Sodium-Glucose Cotransporter 2 Inhibitors vs Sulfonylureas in Patients With Type 2 Diabetes. *JAMA Internal Medicine*. 2021;doi:10.1001/jamainternmed.2021.2488
4. Xie Y, Bowe B, Gibson AK, et al. Comparative Effectiveness of SGLT2 Inhibitors, GLP-1 Receptor Agonists, DPP-4 Inhibitors, and Sulfonylureas on Risk of Kidney Outcomes: Emulation of a Target Trial Using Health Care Databases. *Diabetes Care*. Nov 2020;43(11):2859-2869. doi:10.2337/dc20-1890
5. Xie Y, Bowe B, Gibson AK, et al. Comparative Effectiveness of the Sodium-Glucose Cotransporter 2 Inhibitor Empagliflozin Versus Other Antihyperglycemics on Risk of Major Adverse Kidney Events. *Diabetes Care*. Nov 2020;43(11):2785-2795. doi:10.2337/dc20-1231
6. van Buuren S. Multiple imputation of discrete and continuous data by fully conditional specification. *Statistical methods in medical research*. 2007;16(3):219-242. doi:10.1177/0962280206074463
7. Harrell FE. *Regression modeling strategies: with applications to linear models, logistic and ordinal regression, and survival analysis*. Springer Series in Statistics. Springer, New York, NY.

© 2026 Choi T et al. *JAMA Network Open*.
