# Supplementary material for: GLP-1 Receptor Agonists or SGLT2 Inhibitors and Nonarteritic Anterior Ischemic Optic Neuropathy
Source: JAMA Netw Open. 2026 Apr 30;9(4):e269917. doi: 10.1001/jamanetworkopen.2026.9917 (PMC13133691; doi:10.1001/jamanetworkopen.2026.9917)
Supplement: Supplement 2. — Data Sharing Statement [file jamanetwopen-e269917-s002.pdf]

Data Sharing Statement

Choi. GLP-1 Receptor Agonists or SGLT2 Inhibitors and Nonarteritic Anterior Ischemic Optic Neuropathy. *JAMA Netw Open*. Published April 30, 2026.
doi:10.1001/jamanetworkopen.2026.9917

Data
Data available: No

Additional Information
Explanation for why data not available: Data are available through the United States Department of Veterans Affairs.
